# PrEP in Europe – expectations, opportunities and barriers

**DOI:** 10.7448/IAS.19.7.21103

**Published:** 2016-10-18

**Authors:** Sheena Mary McCormack, Veronica Noseda, Jean-Michel Molina

**Affiliations:** 1 Medical Research Council Clinical Trials Unit, University College London, London, UK; 2 Mouvement Français pour le Planning Familial, Paris, France; 3 Department of Infectious Diseases, Saint-Louis Hospital, and University of Paris Diderot, Sorbonne Paris Cité; 4 INSERM U941, Paris, France

**Keywords:** health systems, Europe, MSM, PWID, migrants

## Abstract

**Introduction:**

In contrast to the global trend showing a decline in new HIV infections, the number reported in the World Health Organization (WHO) region of Europe is increasing. Health systems are disparate, but even countries with free access to screening and treatment observe continuing high rates of new infections in key populations, notably men who have sex with men (MSM). Pre-exposure prophylaxis (PrEP) is only available in France. This commentary describes the European epidemics and healthcare settings where PrEP could be delivered, how need might be estimated for MSM and the residual barriers to access.

**Discussion:**

Health systems and government commitment to HIV prevention and care, both financial and political, differ considerably between the countries that make up Europe. A common feature is that funds for prevention are a small fraction of funds for care. Although care is generally good, access is limited in the middle-income countries of Eastern Europe and central Asia, and only 19% of people living with HIV received antiretroviral therapy in 2014. It is challenging to motivate governments or civil society to implement PrEP in the context of this unmet treatment need, which is driven by limited national health budgets and diminishing assistance from foreign aid. The high-income countries of Western Europe have hesitated to embrace PrEP for different reasons, initially due to key gaps in the evidence. Now that PrEP has been shown to be highly effective in European MSM in two randomized controlled trials, it is clear that the major barrier is the cost of the drug which is still on patent, although inadequate health systems and diminishing investment in civil society are also key challenges to overcome.

**Conclusions:**

The momentum to implement PrEP in European countries is increasing and provides a welcome opportunity to expand and improve clinical services and civil society support focused on HIV and related infections including other sexually transmitted and blood-borne infections.

## Introduction

In contrast to sub-Saharan Africa, estimates of HIV incidence have not decreased in the World Health Organization (WHO) region of Europe. Indeed they increased, and in 2014 the highest number and rate of HIV infections were reported [1]. The majority (77%) were reported from the East (15 countries). Even though numbers appear “stable” in Western Europe (23 countries), this disguises high and rising incidence in subpopulations of men who have sex with men (MSM) [2] confirmed in two recent studies [3,4]. The epidemic in Eastern Europe differs substantially from the West; only 2% of new cases are in MSM, and access to treatment remains a major obstacle to infection control [2]. People who inject drugs (PWID) accounted for 3 and 28% of new diagnoses in Western and Eastern Europe respectively, in 2014. Although outbreaks have been observed in the West and Centre (15 countries), for example in Greece and Romania, they have been rapidly controlled by harm reduction interventions including needle exchange and opiate substitution therapy [5]. This commentary describes the European epidemics and healthcare settings where PrEP could be delivered, how need might be estimated for MSM and the residual barriers to access.

### European epidemic

In 2004, representatives of 53 countries that constitute Europe as defined by the WHO, including the 31 countries that make up the single market of the European Economic Area, met in Dublin and issued a declaration of partnership to fight HIV/AIDS in Europe and central Asia. Part of the declaration was an agreement to monitor progress on the 33 actions to be taken, in alternate years from 2006. These progress reports, together with routine national surveillance data, enable the WHO and the European Centre for Disease Prevention and Control (ECDC) to generate a picture of the regional epidemics, the national responses which depend to a substantial degree on the national economy and the residual challenges.

In 2014, there were 142,197 new diagnoses made in 50 of the 53 countries, the highest annual number since reporting started in the 1980s [2]. Of these diagnoses, 56,945 were officially reported by 49 countries to ECDC and a further 85,252 were reported by the Russian Federal Scientific and Methodological Centre for Prevention and Control of AIDS. The variation in epidemic patterns is considerable across the region with the most striking differences among the 23 countries that make up Western Europe and the 15 countries that make up Eastern Europe. The epidemic change in the West is most apparent among MSM. There has been a sustained increase in estimated incidence of HIV and other sexually transmitted infections (STIs) in this population since 2005, even in countries with good access to treatment and care [6,7]. During the same period, new diagnoses due to the second most common route, heterosexual transmission, declined. However, this was not due to a decline in heterosexual HIV acquired within Western Europe but rather the 52% decline in cases that had acquired their HIV outside the region. Nearly half of those living with HIV present with a CD4 count <350 at diagnosis, and this underscores the need to expand and promote HIV testing services to improve uptake of regular testing in key populations and strengthen linkage to care [2]. In contrast to the countries of Western Europe, the change in the epidemic between 2005 and 2014 is most apparent in females in Eastern Europe, where the two largest countries are Russia and Ukraine. Rates in women have increased by 74% compared with a 49% increase in cases of men. Although women are more susceptible to HIV for biological and sociological reasons (no independent income and domestic violence), this does not entirely explain the gender difference, especially as a substantial proportion of new diagnoses in heterosexual men may be misclassified as MSM and PWID. The higher rates reported in women in this region may represent the “second wave” of infections from a predominantly male population of injecting drug users.

It is important to recognize that the surveillance data do not provide an accurate estimate of the incidence of HIV in subpopulations. In the UK, where the mathematical models of the epidemic are a good fit to the surveillance data, the national estimate of incidence in MSM attending sexual health clinics was 1.6 per 100 person years (PY) in 2014 [8], whereas the observed incidence in the PROUD study participants drawn from the same population was much higher at 9 per 100 PY [3]. Each country has hot spots (geographically) and sexual networks that facilitate HIV transmission. In the IPERGAY trial, for example, HIV incidence in the placebo arm among MSM reporting condomless sex with two or more partners in the previous six months was 9.17 per 100 PY in Paris compared with 2.45 in other large cities (Molina JMM, personal communication). Also, 45% of all newly discovered infections in France in 2014 were diagnosed in the Ile-de-France region, which accounts for only 18% of the overall French population of 66M [9]. These data imply that, even though risk behaviours may be similar, the risk of acquiring HIV infection varies geographically, with MSM living in Paris and the larger Ile-de-France region having a nearly threefold increase in HIV risk acquisition.

The information is most limited for sex workers, trans women, trans men and migrants. Where data do exist, it is clear that the prevalence of HIV is higher than the general population [9–11]. Migrant women account for one in four new diagnoses in France each year but it is not entirely clear where they acquired their HIV and, when in France, whether this was from sex work, or from their partners who may be having sex with men without considering themselves to be gay, or from partners who migrated from countries with high prevalence. Nonetheless, within these populations, the offer of PrEP is likely to appeal most to individuals who recognize their risk, as was the case in PROUD and IPERGAY.

### Service organization

Public health services are highly variable across the region, ranging from open access to free services for HIV and STI testing and treatment through to access only with significant copayments or in the worst-case scenario extremely limited access to non-confidential and pejorative services. Healthcare is funded by the public sector through tax and social insurance contributions in most countries, with a small contribution from private insurance schemes (<5%). In a few countries, including Germany and the Netherlands, healthcare is delivered by the public sector but funded mainly through insurance schemes and/or formal and informal copayments [12]. Regardless of the model, expenditure on health in the European countries that belong to the Organization for Economic Co-operation and Development (OECD), particularly those in Southern Europe, is lagging behind other OECD countries and has been static or shrinking over the last five years, due to the economic crisis [13].

Community-based organizations do offer HIV/STI screening in some settings, frequently tailored to key populations. These services collaborate for post-exposure prophylaxis as antiretroviral prescribing is only available from specialist services.

## Discussion

### Estimating need

The two countries in which the PrEP trials were conducted, France and England, have attempted to estimate the need for PrEP among key populations. In the 2014 French report, there were 6600 new diagnoses of HIV: 42% in MSM (an increase of 5% compared to 2013), 23% among women and 16% among men born in foreign countries. Twenty-one per cent of those from sub-Saharan Africa were thought to have acquired HIV in France. This may be an underestimate, as the ANRS PARCOURS study found that 35% had acquired HIV after migration to France (30% of women and 44% of men) [14]. Hardship was common among migrants from sub-Saharan Africa, with more than 40% living for at least one year without a residence permit and more than 20% with no stable housing. Women who reported hardship were also more likely to report casual and transactional partners. This observation may help services to identify heterosexuals who would benefit from PrEP.

Data from the UK are similar with 6151 new diagnoses in 2014. Although the majority (3360) was in MSM, 1460 heterosexual HIV infections were estimated as acquired in the UK by migrants living in the UK or by those born in the UK. Unfortunately, it is not yet clear how to identify the heterosexuals at risk who would benefit from PrEP. Late presentation among heterosexuals remains unacceptably high and efforts to increase testing in this population are a priority.

Having gathered robust evidence for clinical effectiveness in two randomized controlled trials in MSM, it is possible to identify the characteristics of MSM who would benefit from PrEP. Policy makers have used this information to estimate the likely size of each national PrEP programme to determine the budget impact. In France (66 million inhabitants in 2013), the MSM population is estimated to be around 330,000 persons [15]. In a large anonymous cross-sectional survey conducted in 2011 in France, 20.8% of HIV-negative MSM reported no discernible risk reduction behaviour and can be considered at high risk of HIV acquisition [16]. A seroprevalence survey in Paris found 17.7% of MSM to have HIV, so this suggests that about 50,000 MSM in France may need PrEP [17]. According to the most recent national UK survey of attitudes to sex and lifestyles conducted between 2010 and 2012, 2.6% (95% CI 2.1–3%) of men aged 16 to 74 have had a same sex experience in the preceding five years. Although the majority self-identified as gay, 28% considered themselves to be straight and 19% bisexual [18]. Applying 2.6% to the 2011 UK census estimate of 20 million men aged 15 to 64 suggests that there are 500,000 MSM in this age group. The sexual health clinic network sees 100,000 HIV-negative MSM at least once each year. Behavioural data from clinic surveys (unpublished data, Public Health England) suggest that half or more have had anal sex without a condom in the preceding six months. This generates a similar maximum number to France (50,000) although not all of these individuals may want or need PrEP, as a substantial proportion will be in a monogamous relationship with a concordant negative partner or a positive partner on treatment with undetectable viral load. In the United States PrEP has been available since 2012. Only 49,000 to 80,000 individuals have started PrEP in the United States among an overall population of 323 million with an estimated need among MSM of 492,000 (Grant R, personal communication). Further, a substantial proportion of the early adopters was women. Based on the US experience, a target of 50,000 MSM seems highly aspirational for France and the UK. If 50,000 MSM took PrEP for one year, the budget impact for drug alone would be €150M in the region to support an IPERGAY regimen and almost double to support a daily regimen.

For non-MSM populations, it is less clear who will come forward to access PrEP, what their likely incidence would be without PrEP and how effective PrEP will be. In England, estimated HIV incidence in Black African heterosexuals that access the sexual health clinics is higher than overall heterosexuals (0.17% per year compared to 0.03% in 2012) but still low. About 1000 heterosexual men and 1000 heterosexual women accessed PEP in 2012, and the numbers were similar in 2013.

### Movement in the right direction

The French authorities approved Truvada^®^ (TDF/FTC) under a recommendation for temporary use, effective from 4 January 2016. Truvada is fully covered by the healthcare system but visits and tests will be covered at the usual rate, which is 60% of costs reimbursed. This process is independent of the European Medicines Agency (EMA), was initiated under pressure from civil society and was supported by the Minister of Health for France, who agreed to fully reimburse the costs for drug.

Widespread concern about the possibility that PrEP would lead to a decrease in condom use and precipitate an increase in other STIs inspired the PROUD trial design to compare immediate access to PrEP to a delayed access after 12 months. There were differences in behaviour with a significantly higher proportion of PrEP users reporting 10 or more partners with whom they had had receptive anal sex without a condom in the preceding 90 days (21% immediate PrEP compared to 12% deferred) [3]. However, there were no differences in the proportion who acquired other STIs. In reality, the rates of other STIs have been increasing for the last decade, driven largely by infections in HIV-positive MSM but accompanied by a steady increase in syphilis, gonorrhoea and chlamydia in HIV-negative MSM [6,19]. The introduction of PrEP offers an opportunity to control STIs through regular asymptomatic screening, prompt treatment, and active notification and treatment of partners. Importantly, PROUD demonstrated that the efficacy of PrEP was not undermined by the presence of these other STIs.

The success of the two randomized trials and subsequent implementation of demonstration projects in Amsterdam and in Antwerp have strengthened the partnership between civil society and the medical community in Europe, broadly through the activities of the European AIDS Treatment Group and the EuroPrEP collaboration. The partnership between EATG members and the EuroPrEP clinicians started at the country level around the trials, for example the role of AIDES in the French IPERGAY trial, and the Community Engagement Group that supported PROUD. To date, the European partnership has been concentrated in the countries of Western Europe, but it will be important to expand and support countries in Eastern Europe and central Asia as they embark on demonstration projects or national programmes. A common problem for all countries is the cost of the drug which makes large-scale national PrEP programmes look unaffordable. This is the underlying reason that the PrEP policy has stalled in England and Wales, where the National Health Service is only willing to contribute £2M to the early implementation activities. The EuroPrEP collaboration wrote to Gilead Sciences, the sole source of Truvada^®^ for European governments, on 1 December 2015 with two requests: first that Gilead submit to the EMA as regulatory approval is considered essential for national policy in some countries, and second that they consider reducing the price of drug. Gilead has now submitted to the EMA. While we are not aware of any reductions in the cost of the drug, the company is clearly willing to negotiate at the country level as demonstrated by Portugal and Georgia, where hepatitis C treatment is being fully implemented.

### Residual barriers and solutions

A key challenge for Europe is to meet the needs of other high-risk groups, particularly migrants, for whom the links with community-based organizations and the healthcare system are much lower than for MSM.

While those that purchase health care are concerned that uptake will be higher than planned, advocates and clinicians recognize the reality that many of those in need will not take up the offer of PrEP, especially young MSM aged 15 to 24 – a group in whom the number of new diagnoses has more than doubled since 2003. Their health-seeking behaviours differ considerably from older MSM, and health promotion efforts, as well as services, need to adapt and innovate to meet their needs. An important modification to services will be to build on the partnerships developed during the trials and shift tasks away from clinicians and hospitals towards community-based organizations which are more acceptable venues for individuals who do not consider themselves to be “patients.”

The two major components driving cost-effectiveness are the price of drug and HIV incidence [20]. PrEP is cost saving at the incidence rates reported in the two trials, but barely cost-effective as a daily regimen when the national incidence rates are applied over an 80-year time horizon [21]. The event-driven, on-demand regimen used in IPERGAY and recommended for MSM by the European AIDS Clinical Society [22], utilized about half the amount of drug required to support a daily regimen, equating to a 50% reduction in price. The US Centre for Disease Control and Prevention [23] and WHO [24] do not yet recommend this regimen, but guidelines are likely to be revised as evidence gathers from the European studies. Importantly, TDF/FTC could be available from generic manufacturers in 2017 and European countries should encourage manufacturers to prepare for large-scale demand for this drug, which is also popular as a treatment option.

Assuming the issue of drug costs can be resolved, there are additional requirements to implement a combination prevention strategy incorporating PrEP. This includes raising awareness of PrEP with information campaigns aimed at the “late majority and the laggards,” and building capacity to deliver prevention and care in a more integrated service than currently exists in most countries. This will require political will from government, purchasers and providers of services but could be done by strengthening partnerships and empowering a broader range of providers to undertake screening with minimal additional funding. Governments may need to see a demonstration of these partnerships to be convinced that it is easy to accommodate PrEP within existing reconfigured services. Over the last few years, there has been diminishing investment in civil society and this trend needs to be reversed if we are to effectively raise awareness of PrEP and promote a holistic approach to prevention, which starts with a HIV test. Scaling up and normalizing HIV testing will be critical for countries where HIV has spilled into the general population.

An important starting point for Europe is to strengthen the role and scope of the European Centre for Disease Control and Prevention. This organisation has the data, albeit limited, and is best placed to advise individual countries on the model of prevention and care to adopt.

## Conclusions

There has never been a better time to advocate for strengthening prevention services and increasing access for key populations with increased risk of acquiring HIV and other sexually transmitted and blood-borne infections. These populations are invariably vulnerable, with other health and social care needs. Screening is at the core of this and services need to increase throughput, taking advantage of innovations in self-sampling, self-testing and community-based testing. Governments should be confident of success. With political will, the epidemic trends in Europe could be reversed.
